# Chemical Composition and Antimicrobial Activity of Essential Oil of Fruits from *Vitex agnus-castus* L., Growing in Two Regions in Bulgaria

**DOI:** 10.3390/plants11070896

**Published:** 2022-03-28

**Authors:** Iliya Zhelev, Zhana Petkova, Iliana Kostova, Stanka Damyanova, Albena Stoyanova, Ivanka Dimitrova-Dyulgerova, Ginka Antova, Sezai Ercisli, Amine Assouguem, Mohammed Kara, Rafa Almeer, Amany A. Sayed

**Affiliations:** 1 Faculty of Pharmacy, Medical University of Varna, 9002 Varna, Bulgaria; ijelev80@abv.bg; 2 Department of Chemical Technology, Faculty of Chemistry, University of Plovdiv “Paisii Hilendarski”, 24 Tsar Asen St., 4000 Plovdiv, Bulgaria; zhanapetkova@uni-plovdiv.net (Z.P.); ginant@uni-plovdiv.net (G.A.); 3 Department of Biotechnology and Food Technology, “Angel Kanchev” University of Russe, Razgrad Branch, 7200 Razgrad, Bulgaria; ikostova@uni-ruse.bg (I.K.); sdamianova@uni-ruse.bg (S.D.); 4 Department of Tobacco, Sugar, Vegetable and Essential Oils, University of Food Technologies, 26 Maritza Blvd., 4002 Plovdiv, Bulgaria; aastst@abv.bg; 5 Faculty of Biology, University of Plovdiv “Paisii Hilendarski”, 24 Tsar Asen St., 4000 Plovdiv, Bulgaria; ivadim@uni-plovdiv.bg; 6 Department of Horticulture, Atatürk University, Erzurum 25240, Turkey; 7 Laboratory of Functional Ecology and Environment, Faculty of Sciences and Technology, Sidi Mohamed Ben Abdellah University, Imouzzer Street, Fez 30000, Morocco; assougam@gmail.com; 8 Laboratory of Biotechnology, Conservation and Valorisation of Natural Resources (LBCVNR), Faculty of Sciences, Dhar El Mehraz, University Sidi Mohamed Ben Abdallah, BP 1796 Atlas, Fez 30000, Morocco; mohammed.kara@usmba.ac.ma; 9 Department of Zoology, College of Science, King Saud University, P.O. Box 2455, Riyadh 11451, Saudi Arabia; ralmeer@ksu.edu.sa; 10 Zoology Department, Faculty of Science, Cairo University, Giza 12613, Egypt; amanyasayed@sci.cu.edu.eg

**Keywords:** *Vitex agnus-castus* fruits, chemical composition, essential and vegetable oils, antimicrobial activity

## Abstract

The chemical composition of *Vitex agnus-castus* L. (*Verbenaceae* family) fruits, collected from two regions in Bulgaria (south-central and north-east Bulgaria), was investigated. The content of proteins (5.3–7.4%), carbohydrates (73.9–78.8%), fiber (47.2–49.9%), ash (2.5–3.0%), essential oils (0.5%), and vegetable oil (3.8–5.0%) were identified in the fruits. The composition of the essential oils (EOs) of *Vitex* fruits from both regions was determined; the main compounds were 1,8-cineole (16.9–18.8%), *α*-pinene (7.2–16.6%), sabinene (6.7–14.5%), and bicyclogermacrene (7.3–9.0%), but significant differences in the quantitative and qualitative composition of EOs between the regions were found. The EOs of plants from north-east Bulgaria demonstrated antimicrobial activity against the pathogenic species *Salmonella abony*, *Staphylococcus aureus*, and *Bacillus subtilis*, but the Gram-negative bacteria *Esсherichia coli* and *Pseudomonas aeruginosa* exhibited resistance to the oil. Linoleic acid predominated in vegetable oil from both regions, followed by oleic acid. *β*-sitosterol and *γ*-tocopherol were the main components in the sterol and tocopherol fraction of the lipids. Phosphatidic acids were the main components in the vegetable oil from north-east Bulgaria, while in the vegetable oil from south-central Bulgaria, all phospholipids were found in almost the same quantity. Overall, significant differences were observed in the chemical composition (proteins, carbohydrates, ash and moisture) of the fruits from the two regions of Bulgaria, as well as in the content of the main components of their essential and vegetable oils.

## 1. Introduction

*Vitex agnus-castus* L. belongs to the Verbenaceae family and is a perennial shrub with a strong aromatic odor. The species is native to the Mediterranean region, but in many places of the world grows as an ornamental plant [1]. The plant has been used for more than 2500 years in ancient Greece, Rome and Egypt, and there are applications for different gynecological problems [2].

*Vitex agnus-castus* is a well-known herbal plant (included in the European Pharmacopoeia) which is rich in numerous bioactive substances. A number of studies have been applied to establishing the chemical composition of the essential oils (EOs) obtained from *V. agnus-castus* fruits [3,4]. These show variation in the yield of EOs and their composition, related to the habitat conditions [5,6]. Several authors have reported that the EO yield of different aerial parts of *Vitex* varies; from the fruits it has been found to be 0.21–1% [4,7], and from the leaves 0.35–0.76% [4,7], while from the flowers of *V. pseudo-negundo* (Hausskn.) Hand.-Mzz. the EO yield was reported as ranging from 0.30 to 0.63% [7]. However, the yield of EO from the leaves, flowers and seeds of *V. agnus-castus* has also been reported as 5.5, 6.2, and 11.26%, respectively [5], which is much higher than reported in previous studies.

Several biological activities of *V. agnus-castus* have been demonstrated, including antimicrobial, antifungal, antioxidant, anti-cancer, carminative and sedative activity, as well as ability to treat pre- and post-menstrual disorders, and digestive disorders [2,4,5,6,8,9].

Our previous study on the EOs of *V. agnus-castus* fruit from Bulgaria showed differences in the yield and the composition, related to habitat conditions [6]. It has been suggested that there are two chemotypes, one with a predominance of 1,8-cineole and *α*-pinene, and the other with the presence of mainly (Z)-*β*-farnesene and bicyclogermacrene in the EOs. Most of the studies confirm that one of the main components of *Vitex* fruit EO is 1,8-cineole (11.6–16.13%) [3,4]; the other components, as determined by El Kamari et al. [4], in measurable amounts, are *α*-thujene (9.3%), phyllocladene (8.2%), *α*-pinene (7.9%). Eryigit et al. [3] reported that *trans*-caryophyllene (19.17%) predominated in the fruit’s EOs, followed by sabinene (18.05%), *α*-terpinyl acetate (6.91%), and dihydroselarene (6.73%). Regarding the chemical content of the EOs from leaves, the major components are 1,8-cineole (8.7–18.27%) and caryophyllene (8.6–9.5%) [4,5]. The EO from *V. agnus-castus* has also been demonstrated to have antimicrobial activity against some bacteria, such as *Escherichia coli*, *Pseudomonas aeruginosa*, *Bacillus subtilis*, *Staphylococcus aureus*, *Salmonella typhimurium*, *Enterococcus faecalis*, *Klebsiella pneumonia* and other pathogenic microorganisms [3,4,5]. Furthermore, El Kamari et al. [4] reported that the EO isolated from *Vitex* fruits had better antimicrobial activity against the bacteria compared to the leaf EO. However, this EO was found to be less effective than the control antibiotics used, including ampicillin, ofloxacin and imipenem [3,4].

Studies on *V. agnus-castus* have mainly focused on the chemical composition of its EOs. However, some researchers have investigated the bioactive components of the vegetable oils of the plant. The presence was identified of valuable unsaturated fatty acids, such as linoleic (24.76–54.11%) and oleic acids (16.85–26.11%) in the vegetable oil of *V. agnus-castus* fruits, and sterols (*β*-sitosterol being the main component) in the unsaponifiable fraction [10,11,12]. However, there is a lack of detailed analysis on the composition of both essential and vegetable oils from the fruit of this plant.

The aim of the present study was to supplement and compare the scientific information about the chemical compounds of *V. agnus-castus* fruits (with different sampling period from the fruits of our previous study), growing in two regions in Bulgaria in relation to differences in the soil and climatic conditions of the habitat, and to assess the antimicrobial activity of the derived EOs against different microorganisms. The study also focused on another aspect of the use of *V. agnus-castus* fruits, as a healthy food rich in macronutrients including proteins, carbohydrates and vegetable oil, and abundant in tocopherols, sterols, phospholipids and essential fatty acids. The purpose of this was to evaluate the nutritional value and biological activity of *Vitex* fruits and expedite potential application of the plant in the food and pharmaceutical industries.

## 2. Results

### 2.1. Chemical Composition of the Fruits

The content of total proteins, carbohydrates, fiber, ash and vegetable oil in *V. agnus-castus* L. fruits has not previously been examined. For this reason, the chemical composition (the content of vegetable oil, EOs, proteins, carbohydrates, fiber, ash and moisture) of the fruits from two regions in Bulgaria was investigated (Table 1). In the total carbohydrate content, apart from starch, fiber and available sugars, other components (probably pectin and plant mucilages) were also present which were not determined in the present study. Total carbohydrate content was very high in both samples (73.9 and 78.8%), while the starch content of the fruits from the south-central region of Bulgaria (23.8%) was almost two times higher than that from the north-east region (14.1%). On the other hand, available sugars were found in negligible amounts in the fruits (1.0 and 0.7%). The ash content in the two samples was also low (3.0 and 2.5%, respectively), while their moisture content was found to be 10.7 and 9.6%. Surprisingly, the content of fiber was high in both fruit samples (47.2 and 49.9%). The EOs were characterized as light yellowish liquids with a specific pleasant odor, with a yield of 0.5% v/w for both samples. The vegetable oil and protein content of the fruits were relatively low. The quantity of these compounds in the fruits from the south-central part of Bulgaria (5.0 and 7.4%, respectively) were slightly higher than in in the fruits from the north-east region (3.8 and 5.3%, respectively).

### 2.2. Chemical Composition of the Essential Oils (EOs)

The chemical components of the EOs are listed in Table 2.

Seventeen compounds were common ingredients in oils from the two regions. There were also certain differences in terms of the composition of the main compounds; limonene was identified only in the EOs from the fruits from south-central Bulgaria, while the components present only in the EOs from the north-east region were *β*-(E)-farnesene, alloaromadendrene, and *α*-gurjunene.

The EOs obtained from sample 1 (south-central Bulgaria) included a total of 36 components, representing 98.1% of total oil content, with fifteen in concentrations over 1%. The main components (above 3%) were: 1,8-cineole (18.8%), *α*-pinene (16.6%), sabinene (14.5%), bicyclogermacrene (9.0%), *β*-caryophyllene (6.6%), limonene (5.3%), *tau*-cadinol (3.4%), and *α*-terpinyl acetate (3.3%).

In the EOs from sample 2 (north-east Bulgaria), 36 components were also identified, representing 99.1% of the total content, with 21 in concentrations over 1%. The main components (above 3%) were: 1,8-cineole (16.9%), *β*-caryophyllene (9.0%), *β*-(E)-farnesene (8.0%), bicyclogermacrene (7.3%), *α*-pinene (7.2%), sabinene (6.7%), *α*-terpinyl acetate (6.0%), alloaromadendrene (4.4%), terpinen-4-ol (4.0%), *α*-terpineol (3.9%), and *α*-gurjunene (3.4%).

The distribution of compounds by chemical classes (expressed as a percentage of those identified) is shown in Table 2. Monoterpene hydrocarbons (*α*-pinene and sabinene) were the dominant group in the EOs obtained from the fruits from south-central Bulgaria, followed by oxygenated monoterpenes (1,8-cineole and *α*-terpinyl acetate), sesquiterpene hydrocarbons (bicyclogermacrene and *β*-caryophyllene), oxygenated sesquiterpenes (*tau*-cadinol), aliphatic hydrocarbons, triterpenes, and diterpenes.

Sesquiterpene hydrocarbons (*β*-caryophyllene, bicyclogermacrene, *β*-(E)-farnesene, and alloaromadendrene), were the dominant group in the EOs obtained from the fruits originating from north-east Bulgaria, followed by oxygenated monoterpenes (1,8-cineole, *α*-terpinyl acetate, terpinen-4-ol, and *α*-terpineol), monoterpene hydrocarbons (*α*-pinene, sabinene, and limonene), oxygenated sesquiterpenes (*tau*-cadinol), and phenyl propanoids.

Significant differences were found not only in the main components, but also in the minor constituents of the *Vitex* EOs from the two regions (*p* < 0.05).

### 2.3. Antimicrobial Activity of the Essential Oils (EOs)

The antimicrobial activity of sample 2 was determined. The results are presented in Table 3. The EOs possessed comparatively low antimicrobial potential against the Gram-positive bacteria *Staphylococcus aureus*, *Bacillus subtilis*, and *Kocuria rhizophila*, the Gram-negative bacterium *Salmonella abony*, and the yeast *Saccharomyces cerevisiae*. The Gram-negative bacteria *Esсherichia coli* and *Pseudomonas aeruginosa* were resistant to the inhibitory activity of the investigated EOs.

### 2.4. Biologically Active Components in the Vegetable Oil from Vitex Fruits

The present study was also supplemented by data on the content of the main biologically active components in the fruit vegetable oils from *V. agnus-castus* (Table 4).

Unsaponifiable matter in the oil from sample 2 (north-east Bulgaria) (24.6%) was considerably higher than in the oil from sample 1 (south-central Bulgaria) (16.6%). Total sterols in the oils were 1.4–1.5% and phospholipids were found to be 10.4 and 9.3%. Total tocopherols in the oils were relatively low, but their content in the glyceride oil from the fruits originating from north-east Bulgaria (305 mg/kg) was almost two times higher than in the oil from sample 1 (south-central Bulgaria) (164 mg/kg). It was noticeable that the unsaponifiable matter, total tocopherols and phospholipids differed significantly in the two examined vegetable oils (*p* < 0.05), but the content of total sterols was not influenced by the specific climatic conditions of the regions where the fruits had grown (*p* > 0.05).

Data on the fatty acid composition of the vegetable oils are presented in Table 5.

Nine fatty acids were identified in the oil from sample 1 (south-central Bulgaria), and twelve in the oil extracted from sample 2 (north-east Bulgaria). Analysis of variance followed by Duncan’s multiple range test revealed that significant differences (*p* < 0.05) were observed in the main fatty acids between the two examined vegetable oils, apart from the content of palmitic acid (*p* > 0.05). Linoleic acid predominated in both oils (71.5 and 66.1%, respectively), followed by oleic (14.0 and 16.1%) and saturated palmitic (9.0 and 8.9%) acid. The amount of stearic acid was found to be 3.4 and 4.2%, respectively, while the other fatty acids were present in small quantities (from 0.1 to 1.5%).

The content of saturated, unsaturated, mono- and polyunsaturated fatty acids is also given in Table 5. Unsaturated fatty acids predominated in both oils (85.8 and 84.3%) and the polyunsaturated fatty acids were in a higher quantity (71.5 and 66.7%) than monounsaturated fatty acids (14.3 and 17.6%). Saturated fatty acids were found to be 14.2 (sample 1) and 15.7% (sample 2), respectively. According to the statistics performed, significant differences were observed between the total saturated, mono- and polyunsaturated fatty acids (*p* < 0.05) of the examined vegetable oils from the south-central and north-east regions of Bulgaria.

Sterol, tocopherol and phospholipid profiles of the examined oils are given in Table 6.

## 3. Discussion

Significant differences were observed in the content of moisture, proteins, carbohydrates (including starch, available sugars, fibers), ash and vegetable oil (*p* < 0.05) between the two fruit samples. Apparently, the yield of EOs from the examined fruits was not influenced by the different climatic conditions of the regions where the plants had grown.

The high content of total carbohydrates in both samples influenced the high energy value of the *Vitex* fruits, which was calculated using the conversion factors recommended by the FAO/WHO [13]. The energy value of the fruits from south-central Bulgaria was established to be 1115.3 kJ/100 g, and for those from north-east region of the country, 1121.2 kJ/100 g, which confirmed them to be suitable additives in various food products. These values were lower than the results reported for *V. mollis* Kunth (1433 kJ/100 g) [14], which could be explained by the different content of carbohydrates, proteins and lipids.

The higher EO yield (1%) of the sample from south-central Bulgaria in our previous study [6] compared to the current one, could be explained not only by climatic factors, but also by the sampling period (a month and a half difference) and the degree of maturity of fruits. El Kamari et al. [4] established that the EO yield of *V. agnus-castus* fruits was 1% which was higher than that obtained from the leaves (0.35%). On the other hand, Haghighi et al. [7] reported similar values to the current yield of EOs, but for *V. pseudo-negundo* fruits (0.21–0.45%). They also established that the content of EOs in the fruits was lower than from the other parts of the plant (leaves and flowers) (from 0.33–0.76% and 0.30–0.63%, respectively) [7].

Similar quantities of vegetable oil and proteins in the fruits were observed by Ibrahim et al. [10].

Significant differences were noticed in the chemical composition of the *Vitex* EOs examined in the current investigation compared to the data for our previous study in which the plants had been collected from the same regions, but in September 2013 [6]. Zhelev et al. [6] reported that sabinene (a monoterpene hydrocarbon) was not identified in both EO regional samples collected in 2013, while its quantity in the EOs in the present study was 14.5% and 6.7%, respectively for the sample from the south-central and north-east part of Bulgaria. It was also evident that the EOs from the plants collected in 2013 possessed a higher content of the monoterpene hydrocarbon *β*-pinene (9.4% vs. 1.5% for the samples from south-central Bulgaria and 3.99% vs. 1.2% for the samples from north-east Bulgaria), and a higher amount of the sesquiterpene hydrocarbon *β*-(E)-farnesene (6.88% vs. 0.0% for the samples from south-central Bulgaria and 16.38% vs. 8.0% for the samples from north-east Bulgaria). Some differences were observed in the content of oxygenated monoterpenes as well. For instance, the amount of 1,8-cineole in the EOs from the plants collected in 2013 was 2.96% (from north-east Bulgaria), but its quantity in the EOs from *Vitex* fruits collected in 2018 was much higher (16.9%). On the other hand, the EOs from *V. agnus-castus* plants collected in 2013 were characterized by a very low content of aliphatic hydrocarbons.

The current study did not support the assumption of Zhelev et al. [6] of the presence of two chemotypes of *V. agnus-castus* in Bulgaria. The composition of EOs is influenced by the habitat conditions, the period of collection and the maturity of the fruits, because these are related to chemical changes in the oil composition, which has been pointed out by other authors [3]. Other studies on the composition of the EOs of *V. agnus-castus* fruit have also confirmed variation in the main constituents [3,4,5,8,15,16,17,18,19]. Several of these are defined as major, namely: 1,8-cineole, *β*-caryophyllene, bicyclogermacrene, *α*-pinene, sabinene, terpinen-4-ol, and terpinyl acetate. Ekundayo et al. [20] established that *V. agnus-castus* L. leaf EOs were characterized by extremely high content of 1,8-cineole (50.9%), followed by sabinene (10.8%), *α*-pinene (9.0%), terpinen-4-ol (4.8%), *p*-cymene (4.2%), limonene (2.5%), and *α*-terpineol (2.3%).

The comparative analysis of the EO chemical composition of the two samples examined in the current investigation showed significant differences in the content of the individual groups of the compounds (*p* < 0.05). In the oil from the fruits from south-central Bulgaria (sample 1), the content of monoterpene hydrocarbons was twice as high in comparison to that from north-east Bulgaria (sample 2), and that of sesquiterpene hydrocarbons was twice as low.

In the composition of the EOs from south-central Bulgaria, the major monoterpene hydrocarbons were *α*-pinene (16.6%), sabinene (14.5%), and limonene (5.3%), results which are close to those obtained by Sorensen and Katsiotis [15]. The quantity of the oxygenated monoterpene 1,8-cineole (18.8%) was also high, which was confirmed by Senatore et al. [16]. The content of the sesquiterpene hydrocarbons, bicyclogermacrene (9.0%) and *β*-caryophyllene (6.6%), was also found to be relatively high and similar to that found by Senatore et al. [16].

The EOs of *Vitex* fruits from north-east Bulgaria (sample 2) contained a higher quantity of the oxygenated monoterpene 1,8-cineole (16.9%) and *α*-terpinyl acetate (6.0%), which was similar to the results obtained by Senatore et al. [16]. The fraction of sesquiterpene hydrocarbons in this EO sample contained high amounts of *β*-caryophyllene (9.0%), *β*-(E)-farnesene (8.0%), and bicyclogermacrene (7.3%), which was also close to the findings of Senatore et al. [16].

These differences can be explained by the different climatic conditions in the different regions of the country which affect the biosynthesis and composition of EOs. In the south-central region of Bulgaria (sample 1), the summer is usually characterized by high temperatures and dry weather, which slows down the evaporation of the low molecular weight monoterpene hydrocarbons. Conversely, in the north-eastern region of the country (sample 2), the summer is longer, and more humid because of the influence of the Black Sea, and the higher temperatures provoke the evaporation of the abovementioned components from the oil into the atmosphere. Similar effects on the composition of EOs obtained from fruits harvested from different geographical locations have been found in other plants [21,22].

One of the main ecological roles of EOs, as natural complexes of volatile compounds, produced by plants, is protection against bacteria, viruses and fungi [23]. Therefore, the antimicrobial potential of *Vitex* fruit EOs was also studied. Due to higher amounts of oxygenated derivatives in the EOs from the fruits originating from the north-east region (sample 2), for example, alcohols, such as terpinen-4-ol, *α*-terpineol, spathulenol, cadinol, etc., esters, such as *α*-terpinyl acetate, and oxides, such as caryophyllene oxide and 1,8-cineole, antimicrobial activity was examined only for this sample. It is known that the antimicrobial activity of the main components of EOs is arranged in the following sequence: phenols > alcohols > aldehydes > ketones > esters > hydrocarbons [24].

In a study by Eryigit et al. [3], *Staphylococcus aureus*, *Bacillus subtilis*, and *Salmonella abony* had similarly low sensitivity to the studied fruit oil. However, different results were found with respect to the antibacterial effect of the EOs from *V. agnus-castus* [3,5,8]. The first authors established the most marked activity of the oils against *Staphylococcus aureus*, *Klebsiella pneumonea*, *Esсherichia coli*, and *Pseudomonas aeruginosa*. Ghannadi et al. [8] confirmed resistance to the oil only for *Esсherichia coli* while *Pseudomonas aeruginosa* and *Staphylococcus aureus* were highly sensitive to the oil. These results could be explained by differences in the composition of the oils.

The unsaponifiable matter of the seed oils consisted mainly of terpenic (sterols, tocopherols, tocotrienols, carotenoids, etc.) and aliphatic (fatty alcohols, saturated and unsaturated hydrocarbons) compounds [25].

The contents of unsaponifiable matter in the examined vegetable oils were much higher than those of sunflower (1.5%), rapeseed (2.0%), maize (2.8%), and grapeseed oil (2.0%) [26].

Total tocopherols in *Vitex* fruit oils were found in lower amounts than in some other vegetable oils, such as sesame seed oil (330–1010 mg/kg), sunflower oil (440–1520 mg/kg) and especially soybean oil (600–3370 mg/kg) [26].

The fatty acid composition is an important characteristic of vegetable oils [27]. Caprylic, linolenic, and arachidic acids were not identified in the oil from the south-central part of the country. Similar results for the content of the main fatty acids were observed by Asdadi et al. [27] (linoleic acid—69.75%, oleic—16.41%, palmitic—6.18%, and stearic—4.23%). Ozkaya et al. [11] reported lower levels of linoleic acid in *Vitex* vegetable oil (54.11%), a similar content of oleic (16.85%) and palmitic (8.66%) acids, and much higher content of linolenic acid (6.86%). Nonetheless, completely different results of the fatty acid composition were observed by Ibrahim et al. [10] who found that oleic (26.11%), linoleic (24.76%) and palmitic (21.01%) acids occurred in similar quantities in the vegetable oil.

Other authors support our results [10,11,27] and define *Vitex* vegetable oil as appropriate for consumption having dietetic value because of its fatty acid/total saturated fatty acid ratio [12]. The fatty acid composition of the examined *Vitex* fruits vegetable oils was similar to that of sunflower and safflower oils, where linoleic acid (48.3–74.0% and 67.8–83.2%, respectively) was also the main component, followed by oleic (14.0–39.4% and 8.4–21.3%) and palmitic acid (5.0–7.6% and 5.3–8.0%) [26].

The high amount of linoleic acid makes *Vitex* seed oil specifically prone to oxidation, and at the same time may have beneficial physiological effects in the prevention of both coronary heart disease and cancer [12]. Linoleic acid is also considered to be an essential fatty acid which is not synthesized in the human body and requires to be provided by food [28,29].

A major part of the unsaponifiable matter in the oils consisted of sterols [12]. Significant differences between the sterol composition of the examined fruit vegetable oils were observed (*p* < 0.05). *β*-Sitosterol was the major sterol in both oils (43.3 and 57.1%). The second largest in the fruit oil from the south-central region was Δ^5^-avenasterol (24.0%), while in that from the north-east region of the country was campesterol (21.7%). The content of brassicasterol was relatively high in both oils (12.3 and 12.0%), while stigmasterol was present in low quantities (7.1 and 3.1%). Similar to our results, Asdadi et al. [12] determined that *β*-sitosterol was the main component, followed by stigmasterol. However, in Bulgarian *Vitex* vegetable oils, campesterol and brassicasterol were the second highest in the oil content. A high quantity of Δ^5^-avenasterol was observed in *Vitex* vegetable oil from the southern part of Bulgaria.

*β*-Sitosterol was also found to be a main component in the majority of the vegetable oils, such as olive, soybean, and sunflower oil [30]. For example, the sterol composition of both examined oils was similar to that of rapeseed oil, where *β*-sitosterol comprised 45.1–57.9%, campesterol was 24.7–38.6%, and the content of brassicasterol was found to be from 5.0 to 13.0% [25]. Sterols from vegetable oils have been shown to lower the total and LDL cholesterol levels in humans [31], therefore *Vitex* seed oil sterols may be used as new therapeutic agents for treatment of hypercholesterolemia.

Other valuable compounds from the unsaponifiable part of the oils include tocopherols. The tocopherol composition of the examined oils was also different (*p* < 0.05). γ-Tocopherol was the only tocopherol identified in the oil from north-east Bulgaria. It was also the major component in the oil from the plants from the south-central part of the country (78.7%), followed by α-tocopherol (21.3%). Other similar data on the presence of tocopherols indicated the low content of the latter (18.20 μg/g) in *Vitex* seed oil [11]. Tocopherol composition of vegetable oils from *Vitex* fruit was significantly different from other commonly used vegetable oils, such as sunflower (where α-tocopherol predominated), soybean (where γ- and δ-tocopherols were the main components), and corn oils (where γ-tocopherol predominated, but there was presence of measurable amounts of α- and δ-tocopherols), and was rather similar to that of sesame oil (where γ-tocopherol was also the major component) [26].

Phospholipids are polar lipids which play an important role in cell membranes, and can be considered as antioxidants because of their metal scavenging activity [32]. The presence of phospholipids is related to the prevention of different diseases and symptoms, e.g., coronary heart disease, inflammation and cancer [33]. The phospholipid content of vegetable oils from *Vitex* fruits was examined for the first time. There were slight differences in the content of phospholipids in the two investigated fruit vegetable oils (*p* < 0.05). Phosphatidic acids were the main component in the sample from north-east Bulgaria (23.7%), while in the sample from the south-central region, phosphatidylinositol (18.3%), predominated, followed by phosphatidylethanolaminе (17.1%) and phosphatidic acid (16.8%). phosphatidylethanolaminе and phosphatidylinositol were the other compounds in the fruits from the north-east part of the country with relatively high concentrations (16.7 and 14.1%, respectively). The content of other phospholipid components varied from 10.7% to 12.6%. The differences in the phospholipid composition in the observed samples of *V. agnus-castus* were probably due to the regions where they had been grown, reflecting the temperature and rate of precipitation in both areas.

Overall, slight differences were observed in the individual composition of sterols, tocopherols and phospholipids of the vegetable oils from the two samples of *Vitex* fruits, which was probably due to the specific differences between the regions where the plants had grown.

## 4. Materials and Methods

### 4.1. Plant Collection

**Figure 1:**
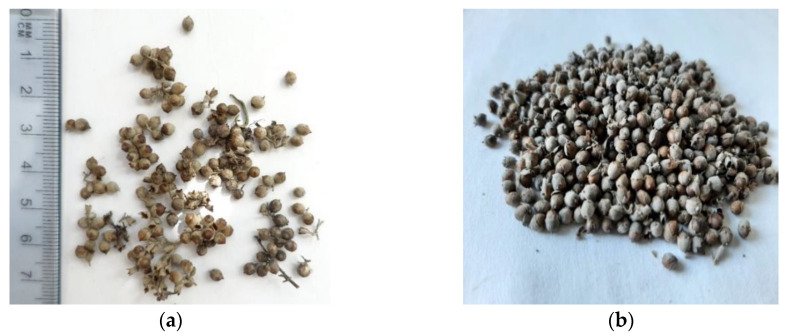
Fruits from *V. agnus-castus* L. (authors’ images). (**a**) Fruits from south-central region of Bulgaria. (**b**) Fruits from north-east region of Bulgaria.

The fruits of *V. agnus-castus* L. were collected from two regions of Bulgaria (Plovdiv town from south-central part of Bulgaria (160 m elev., 42°08′270″ N and 24°47′28″ E), named as sample 1, and Varna region north-east Bulgaria, village Bliznaci (80 m elev., 43°04′43″ N and 27°51′38″ E), named as sample 2), in November 2018 (Figure 1).

The samples were taken from the same places and individual plants on which our previous study was performed [6], when the species were identified and voucher specimens (№ 060437, 060438) were deposited in the herbarium of the Agricultural University, Plovdiv.

The fruits were placed in paper sacks and stored in a cool, dry, well-ventilated, dark room.

### 4.2. Chemical Composition of the Fruits

Protein content, crude fiber, ash and moisture were determined using methods described in AOAC [34]. The following formula was used to calculate total carbohydrates: 100 − (weight in grams (protein + lipids + water + ash) in 100g of dry fruits) [13]. The soluble carbohydrates and the starch content were identified using BS 7169 [35] and BS 13488 [36].

### 4.3. Isolation of the Essential Оil (ЕО)

The fruits were subjected to hydrodistillation for 3 h in a laboratory glass apparatus as described in the British Pharmacopoeia, modified by Balinova and Diakov [37]. The EO was dried over anhydrous sodium sulfate and stored at 4 °C in dark vials until analysis.

After hydrodistillation the fruits were air dried (moisture 7.00% ± 0.06) for 10 days at room temperature (25 °C ± 1).

### 4.4. Chemical Composition of Essential Oil (EO)

The chemical composition of the EOs was determined by gas chromatography (GC) analysis using gas chromatograph Agilent 7890A (Santa Clara, CA, USA), with HP-5 ms column (30 m × 250 µm × 0.25 µm). The temperature program used for the analysis was: 35 °C/3 min, 5 °C/ min to 250 °C for 3 min, total time: 49 min. The carrier gas was helium with a constant speed of 1 mL/min; the split ratio was 30:1. The GC/MS analysis was performed on a mass spectrometer Agilent 5975C, with helium carrier gas. The column and the temperature program were the same as for the GC analysis. The identification of the chemical compounds was performed based on their retention time and library data. The identified constituents were arranged in order of their retention time and their quantity was recorded in percentages.

### 4.5. Antimicrobial Activity of Essential Oil (EO)

Antimicrobial activity of the EOs was tested against the following test microorganisms: Gram-positive bacteria *Staphylococcus aureus* ATCC 6538, *Bacillus subtilis* ATCC 6633, *Kocuria rhizophila* ATCC 9341; Gram-negative bacteria *Esсherichia coli* ATCC 8739, *Pseudomonas aeruginosa* ATCC 9027, *Salmonella abony* NTCC 6017; and yeast, *Saccharomyces cerevisiae* ATCC 2601. Test microorganism strains were supplied by the National Bank for Industrial Microorganisms and Cell Cultures, Sofia, Bulgaria.

The antimicrobial activity was determined by the agar well diffusion method. The growth media were Tryptic soy agar (Merck) for tested bacteria, and Sabouraud-Dextrose-Agar (Merck KGaA, Darmstadt, Germany) for yeast. The media were inoculated with 24 h suspension of the bacterial species and 48 h of the yeast with turbidity—0.5 McFarland standard. Media, melted and cooled to 50 °C ± 2 °C, were inoculated with 1% of the prepared suspensions of the test microorganisms. Quantities of 20 mL of inoculated media were poured into sterile petri dishes (∅ = 90 mm). The agar plates were allowed to solidify. A cork-borer was used to punch holes (∅ = 8 mm) in the agar. A solution of EO in dimethyl sulfoxide (1:10, *v*/*v*) was prepared and 50 μL was added dropwise to each well. Then, the petri dishes were placed in thermostatic chambers and incubated at 37 °C or 28 °C for 24 and 48 h, according to the microbial species. After cultivation, the zone of growth inhibition around the wells was measured using a digital caliper. The diameter of zones, including the diameter of the well, was recorded in mm. Result analyses were interpreted in terms of the diameter of the inhibition zone: up to 15 mm microbial culture was defined as poorly sensitive, from 15 to 25 mm, sensitive and over 25 mm, very sensitive. The tests were performed in parallel with solvent controls [38].

### 4.6. Isolation of Vegetable Oil

The vegetable oil was extracted from ground fruits with *n*-hexane using a Soxhlet extractor [39].

#### 4.6.1. Fatty Acid Composition

GC was used for determination of the fatty acid composition of triacylglycerols [40]. Briefly, the triacylglycerols were pre-esterified with methanol in the presence of sulfuric acid in order to obtain fatty acid methyl esters (FAMEs) [41]. Determination of FAMEs was carried out on an HP 5890 gas chromatograph (Santa Clara, CA 95051, USA) equipped with a capillary column Supelco (75 m × 0.18 mm × 25 μm (film thickness)) (St. Louis, MO, USA) and a flame ionization detector (FID). For the identification of the FAMEs, a standard mixture Supelco, USA (FAME mix 37 components, FAME mix—Sigma Aldrich, Darmstadt, Germany) was used.

#### 4.6.2. Determination of Sterols

Unsaponifiables were determined according to ISO standard [42]. The sterols were isolated from the unsaponifiable matter by thin-layer chromatography (TLC) [43] and their total content was determined spectrophotometrically at a wavelength of 597 nm. Individual sterol composition was determined on an HP 5890 gas chromatograph (Santa Clara, CA, USA) equipped with DB—5 capillary column (25 m × 0.25 mm) (Santa Clara, CA 95051, USA) and FID. Identification was established by comparing the retention times with those of a standard sterol mixture (Across Organics, Morris Plains NJ, USA) [44].

#### 4.6.3. Determination of Tocopherols

Individual tocopherols were determined by high performance liquid chromatography (HPLC) Merck-Hitachi (Merck, Darmstadt, Germany). The column was Nucleosil Si 50-5 (250 mm × 4 mm). Fluorescent detection was used (excitement at 295 nm and emission at 330 nm). The mobile phase used was *n*-hexane:dioxane, 96:4 (*v*/*v*) and the flow rate was set at 1 mL/min [45].

#### 4.6.4. Determination of Phospholipids

Ground fruits were subjected to Folch extraction [46]. For the isolation of the individual phospholipid classes two-dimensional TLC was used [47]. The identified phospholipid spots were scraped and mineralized with perchloric and sulfuric acid, 1:1 (*v*/*v*). The amount of the different phospholipids was determined spectrophotometrically at 700 nm [48].

### 4.7. Statistics

The analyses were performed in triplicate. The results are given as mean ± standard deviation (SD). Statistical significance was determined by one-way ANOVA (Duncan’s test with a significance level of *p* < 0.05) using IBM SPSS Statistics 19.

## 5. Conclusions

The quantity of carbohydrates (starch and sugars), as well as fiber and ash, were investigated for the first time in this study. The composition of the essential oils from fully mature fruits of *V. agnus-castus* from two regions of Bulgaria was represented by the following common main components: 1,8 cineole, *α*-pinene, sabinene, *β*-caryophyllene, bicyclohermacrine and terpinyl acetate, among which 1,8-cineole was the best represented compound in both essential oils. The essential oil of the fruits from the north-east region of Bulgaria demonstrated antimicrobial activity against Gram-positive bacteria *Salmonella abony*, *Staphylococcus aureus*, and *Bacillus subtilis*, but the Gram-negative bacteria *Esсherichia coli* and *Pseudomonas aeruginosa* exhibited resistance to the oil. The main fatty acids in the vegetable oils of the samples were linoleic, oleic and palmitic acid. *β*-Sitosterol was the major sterol in both vegetable oils, and *γ*-tocopherol predominated in the tocopherol fraction. The phospholipid profile from *Vitex* vegetable oil was also studied for the first time. Phosphatidic acids were the main component in the vegetable oil from north-east Bulgaria, while in the vegetable oil from south-central Bulgaria, phosphatidylinositol, phosphatidylethanolamine and phosphatidic acids predominated. Differences in antimicrobial activity against pathogenic microorganisms between our study and those of other authors were evident, which indicates a need for more in-depth investigation of the effects of *Vitex* fruit substances.

## Figures and Tables

**Table 1:** Chemical composition of the fruits ^1^.

Content, %	Sample 1 (South-Central Bulgaria)	Sample 2 (North-East Bulgaria)
Moisture	10.7 ± 0.2 ^a,2^	9.6 ± 0.1 ^b^
Proteins	7.4 ± 0.1 ^a^	5.3 ± 0.1 ^b^
Carbohydrates	73.9 ± 0.7 ^a^	78.8 ± 0.8 ^b^
starch	23.8 ± 0.2 ^a^	14.1 ± 0.1 ^b^
available sugars	1.0 ± 0.1 ^a^	0.7 ± 0.0 ^b^
fiber	47.2 ± 0.5 ^a^	49.9 ± 0.5 ^b^
Ash	3.0 ± 0.2 ^a^	2.5 ± 0.1 ^b^
Essential oil	0.5 ± 0.1 ^a^	0.5 ± 0.0 ^a^
Vegetable oil	5.0 ± 0.1 ^a^	3.8 ± 0.2 ^b^

^1^ Mean ± SD (*n* = 3). ^2^ Values with different letters in the same row indicate significant differences (*p*  ˂  0.05) using Duncan’s test.

**Table 2:** Chemical composition of EOs of *V. agnus-castus* fruits ^1^.

No.	Compounds, %	RI	Sample 1 (South-Central Bulgaria)	Sample 2 (North-East Bulgaria)
1.	*α*-Thujene	931	0.2 ± 0.0	- ^2^
2.	*α*-Pinene	939	16.6 ± 0.15 ^b,3^	7.2 ± 0.1 ^a^
3.	Camphene	954	0.3 ± 0.0	-
4.	Sabinene	971	14.5 ± 0.1 ^b^	6.7 ± 0.1 ^a^
5.	*β*-Pinene	979	1.5 ± 0.0 ^b^	1.2 ± 0.0 ^a^
6.	*β*-Myrcene	991	2.6 ± 0.0 ^b^	2.1 ± 0.0 ^a^
7.	*α*-Phellandrene	1003	1.9 ± 0.0 ^b^	0.9 ± 0.0 ^a^
8.	*α*-Terpinene	1014	0.3 ± 0.0 ^b^	0.6 ± 0.0 ^a^
9.	*p*-Cymene	1022	-	1.2 ± 0.0
10.	Limonene	1029	5.3 ± 0.1	-
11.	1,8-Cineole	1032	18.8 ± 0.2 ^b^	16.9 ± 0.2 ^a^
12.	*γ*-Terpinene	1055	1.4 ± 0.0 ^b^	1.2 ± 0.0 ^a^
13.	cis-Sabinene hydrate	1065	0.2 ± 0.0	-
14.	Terpinolene	1080	-	0.5 ± 0.0
15.	*β*-Linalool	1096	0.5 ± 0.0 ^a^	0.5 ± 0.0 ^a^
16.	*cis*-*p*-Menth-2-en-1-ol	1118	-	0.2 ± 0.0
17.	*trans*-*p*-Menth-2-en-1-ol	1136	-	0.2 ± 0.0
18.	Terpinen-4-ol	1179	1.2 ± 0.0 ^b^	4.0 ± 0.0 ^a^
19.	*α*-Terpineol	1189	2.0 ± 0.0 ^b^	3.9 ± 0.0 ^a^
20.	*β*-Citronellol	1208	-	0.3 ± 0.0
21.	Nerol	1227	-	0.2 ± 0.0
22.	Geraniol	1249	-	0.2 ± 0.0
23.	*δ*-Terpinyl acetate	1316	-	0.3 ± 0.0
24.	*α*-Terpinyl acetate	1333	3.3 ± 0.0 ^b^	6.0 ± 0.1 ^a^
25.	*β*-Elemene	1390	0.5 ± 0.0	-
26.	*α*-Gurjunene	1409	-	3.4 ± 0.0
27.	*β*-Caryophyllene	1429	6.6 ± 0.1 ^b^	9.0 ± 0.1 ^a^
28.	(Z)-Farnesene	1442	-	1.0 ± 0.0
29.	*β*-(E)-Farnesene	1454	-	8.0 ± 0.1
30.	*α*-Caryophyllene	1456	-	0.6 ± 0.0
31.	Alloaromadendrene	1461	-	4.4 ± 0.0
32.	Germacrene D	1484	0.6 ± 0.0	-
33.	Elixene	1492	-	1.11 ± 0.0
34.	Bicyclogermacrene	1501	9.0 ± 0.1 ^b^	7.3 ± 0.1 ^a^
35.	*γ*-Cadinene	1513	-	0.4 ± 0.0
36.	*δ*-Cadinene	1522	-	0.5 ± 0.0
37.	Caryophyllene oxide	1574	0.5 ± 0.0 ^b^	1.4 ± 0.0 ^a^
38.	Globulol	1590	-	0.3 ± 0.0
39.	Ledol	1602	2.0 ± 0.0 ^b^	1.1 ± 0.0 ^a^
40.	(-)-Spathulenol	1619	0.5 ± 0.0 ^b^	2.4 ± 0.0 ^a^
41.	*tau*-Cadinol	1628	3.4 ± 0.0 ^b^	2.2 ± 0.0 ^a^
42.	*α*-Cadinol	1641	-	1.7 ± 0.0
43.	β-Eudesmol	1642	0.1 ± 0.0	-
44.	n-Heptadecane	1700	0.2 ± 0.0	-
45.	n-Heneicosane	2100	0.2 ± 0.0	-
46.	Phytol	2105	0.3 ± 0.0	-
47.	n-Docosane	2200	0.4 ± 0.0	-
48.	n-Tricosane	2300	0.4 ± 0.0	-
49.	n-Tetracosane	2400	0.2 ± 0.0	-
50.	n-Pentacosane	2500	0.2 ± 0.0	-
51.	n-Hexacosane	2600	0.4 ± 0.0	-
52.	n-Heptacosane	2700	0.7 ± 0.0	-
53.	Octacosane	2800	0.6 ± 0.0	-
54.	Squalene	2817	0.7 ± 0.0	-
Aliphatic hydrocarbons, %			3.5	-
Monoterpene hydrocarbons, %			45.5	20.5
Oxygenated monoterpene, %			26.5	32.7
Sesquiterpene hydrocarbons, %			17.0	36.5
Oxygenated sesquiterpenes, %			6.5	9.1
Diterpenes, %			0.3	-
Triterpenes, %			0.7	-
Phenyl propanoids, %			-	1.2

^1^ Mean ± SD (*n* = 3). ^2^ Not identified. ^3^ Values with different letters in the same row indicate significant differences (*p*  ˂  0.05) using Duncan’s test.

**Table 3:** Antimicrobial activity of EOs from *Vitex* fruits (sample 2 from north-central Bulgaria) ^1,2^.

Tested Microorganisms	Inhibition Zone (mm) ^3,4^	
*Staphylococcus aureus* ATCC 6538	11.25 ± 0.05	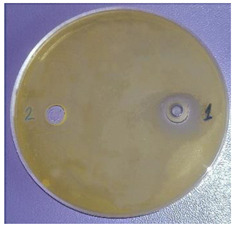
*Bacillus subtilis* ATCC 6633	12.03 ± 0.02	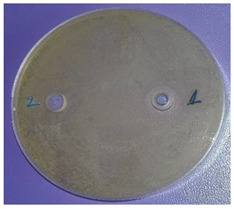
*Kocuria rhizophila* ATCC 9341	9.37 ± 0.04	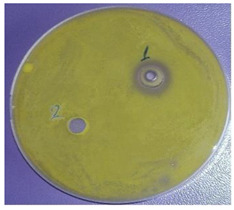
*Escherichia coli* ATCC 8739	8.00 ± 0.0	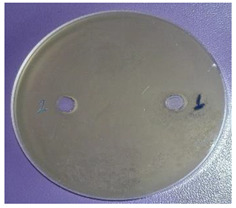
*Pseudomonas aeruginosa* ATCC 9027	8.03 ± 0.02	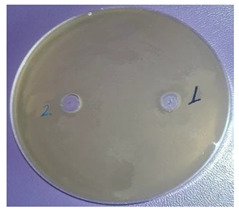
*Salmonella abony* NCTC 6017	11.15 ± 0.05	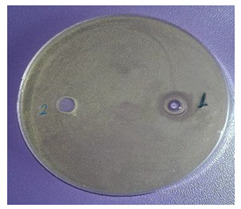
*Saccharomyces cerevisiae* ATCC 2601	11.86 ± 0.03	

^1^ Mean ± SD (*n* = 3). ^2^ The antimicrobial activity of the EOs from the sample from south-central Bulgaria was not determined due to the low amounts of oxygenated derivatives. ^3^ (1) EOs from *V. agnus-castus* L. fruits; (2) solvent. ^4^ Figure is representative of three replicates.

**Table 4:** Total content of unsaponifiable matter, sterols, phospholipids and tocopherols in the vegetable oils from the fruits ^1^.

Biologically Active Components	Sample 1 (South-Central Bulgaria)	Sample 2 (North-East Bulgaria)
Unsaponifiable matter, %		
in the oil	16.6 ± 0.1 ^a,2^	24.6 ± 0.2 ^b^
in the fruits	0.8 ± 0.0 ^a^	0.9 ± 0.0 ^b^
Sterols, %		
in the oil	1.4 ± 0.1 ^a^	1.5 ± 0.2 ^a^
in the fruits	0.1 ±0.0 ^a^	0.1 ± 0.0 ^a^
Phospholipids, %		
in the oil	10.4 ± 0.1 ^a^	9.3 ± 0.1 ^b^
in the fruits	0.5 ± 0.0 ^a^	0.4 ± 0.0 ^b^
Tocopherols, mg/kg		
in the oil	164 ± 2 ^b^	305 ± 3 ^a^
in the fruits	8.1 ± 0.1 ^b^	11.6 ± 0.1 ^a^

^1^ Mean ± SD (*n* = 3). ^2^ Values with different letters in the same row indicate significant differences (*p*  ˂  0.05) using Duncan’s test.

**Table 5:** Fatty acid composition of fruit vegetable oils ^1^.

Fatty Acids, %		Sample 1 (South-Central Bulgaria)	Sample 2 (North-East Bulgaria)
Caprylic	C _8:0_	- ^2^	0.1 ± 0.0
Capric	C _10:0_	0.8 ± 0.2 ^a,4^	0.3 ± 0.1 ^b^
Lauric	C _12:0_	0.3 ± 0.0 ^a^	0.1 ± 0.0 ^b^
Myristic	C _14:0_	0.5 ± 0.1 ^a^	0.2 ± 0.0 ^b^
Palmitic	C _16:0_	9.0 ± 0.2 ^a^	8.9 ± 0.1 ^a^
Palmitoleic	C _16:1_	0.3 ± 0.0 ^a^	1.5 ± 0.1 ^b^
Margaric	C _17:0_	0.2 ± 0.0 ^a^	0.4 ± 0.0 ^b^
Stearic	C _18:0_	3.4 ± 0.2 ^a^	4.2 ± 0.3 ^b^
Oleic	C _18:1_	14.0 ± 0.1 ^a^	16.1 ± 0.1 ^b^
Linoleic	C _18:2_	71.5 ± 0.7 ^a^	66.1 ± 0.6 ^b^
Linolenic	C _18:3_	-	0.6 ± 0.0
Arachidic	C _20:0_	-	1.5 ± 0.1
SFA ^3^		14.2	15.7
UFA		85.8	84.3
MUFA		14.3	17.6
UFA		71.5	66.7

^1^ Mean ± SD (*n* = 3). ^2^ Not identified. ^3^ SFA—saturated fatty acids; UFA—unsaturated fatty acids; MUFA—monounsaturated fatty acids; PUFA—polyunsaturated fatty acids. ^4^ Values with different letters in the same row indicate significant differences (*p * ˂  0.05) using Duncan’s test.

**Table 6:** Sterol, tocopherol and phospholipid composition of fruit vegetable oils ^1^.

Compounds, %	Sample 1 (South-Central Bulgaria)	Sample 2 (North-East Bulgaria)
**Sterols**		
Brassicasterol	12.3 ± 0.1 ^a,3^	12.0 ± 0.1 ^b^
Campesterol	13.3 ± 0.1 ^a^	21.7 ± 0.2 ^b^
Stigmasterol	7.1 ± 0.2 ^a^	3.1± 0.1 ^b^
*β*-Sitosterol	43.3 ±0.4 ^a^	57.1± 0.6 ^b^
Δ^5^-Avenasterol	24.0 ± 0.2 ^a^	6.1± 0.1 ^b^
**Tocopherols**		
*α*-Tocopherol	21.3 ± 0.2	- ^2^
*γ*-Tocopherol	78.7 ± 0.8 ^a^	100 ± 0.0 ^b^
**Phospholipids**		
Phosphatidylinositol	18.3 ± 0.2 ^a^	14.1 ± 0.1 ^b^
Phosphatidylcholine	14.3 ± 0.1 ^a^	12.6 ± 0.1 ^b^
Phosphatidylethanolamine	17.1 ± 0.2 ^a^	16.7 ± 0.2 ^a^
Phosphatidic acids	16.8 ± 0.2 ^a^	23.7 ± 0.3 ^b^
Phosphatidylserine	11.1 ± 0.1 ^a^	10.7 ± 0.1 ^b^
Lysophosphatidylethanolamine	11.0 ± 0.2 ^a^	11.2 ± 0.1 ^a^
Lysophosphatidylcholine	11.4 ± 0.1 ^a^	11.0 ± 0.2 ^b^

^1^ Mean ± SD (*n* = 3). ^2^ Not identified.^3^ Values with different letters in the same row indicate significant differences (*p*  ˂  0.05) using Duncan’s test.

## Data Availability

Data is contained within the article.
